# VES: A Mixed-Reality Development Platform of Navigation Systems for Blind and Visually Impaired

**DOI:** 10.3390/s21186275

**Published:** 2021-09-18

**Authors:** Santiago Real, Alvaro Araujo

**Affiliations:** B105 Electronic Systems Lab, Escuela Técnica Superior de Ingenieros de Telecomunicación, Universidad Politécnica de Madrid, Avenida Complutense 30, 28040 Madrid, Spain; araujo@b105.upm.es

**Keywords:** mixed reality, virtual reality, assistive technology, visually impaired, navigation system, electronic travel aid, wireless sensor actuator network

## Abstract

Herein, we describe the Virtually Enhanced Senses (VES) system, a novel and highly configurable wireless sensor-actuator network conceived as a development and test-bench platform of navigation systems adapted for blind and visually impaired people. It allows to immerse its users into “walkable” purely virtual or mixed environments with simulated sensors and validate navigation system designs prior to prototype development. The haptic, acoustic, and proprioceptive feedback supports state-of-art sensory substitution devices (SSD). In this regard, three SSD were integrated in VES as examples, including the well-known “The vOICe”. Additionally, the data throughput, latency and packet loss of the wireless communication can be controlled to observe its impact in the provided spatial knowledge and resulting mobility and orientation performance. Finally, the system has been validated by testing a combination of two previous visual-acoustic and visual-haptic sensory substitution schemas with 23 normal-sighted subjects. The recorded data includes the output of a “gaze-tracking” utility adapted for SSD.

## 1. Introduction

Visual impairment has an estimated prevalence of 217 million people worldwide, out of which 36 million are blind [1]. This condition heavily impacts an individuals’ autonomy and quality of life even in simple daily routines. Therefore, it motivated the pursuit of new tools which could make up for the missing sensory input, at least partially and in specific tasks.

Particularly, a navigation system for blind and visually impaired people (BVIP) is a device which provide(s) its user with useful and/or required data to reach a destination point [2]. This ranges from simple left/right indications to raw distance measurements, e.g., encoded as haptic stimuli intensity triggered by actuators. For the last few years, we observed an unprecedent growth in this field: guidance systems based on Bluetooth beacon networks or Global Navigation Satellite Systems (GNSS), e.g., [3,4,5]; artificial intelligence systems that recognizes key features of camera images ([6]); or even general-purpose SSD (e.g., [7,8]) have been developed, some of which are currently available as free smartphone applications ([2]).

However, full autonomy in orientation and mobility tasks have not been achieved yet, and further progress is being hampered by technical and theoretical challenges that vary according to the specific application [2]. Some of them are related to the following points:

- Environmental monitoring. The difficulty lies in increasing the set of variables to identify, e.g., obstacles in the user’s path, landmarks, etc., while maintaining the reliability needed for mobility and orientation assistance [2].
- Human-machine interface. The information that can be provided to the user is heavily restricted by the maximum data throughput of the remaining sensory modalities. Furthermore, exploiting these sensory modalities may result in an excessive cognitive load, and masks natural feedback of the environment, e.g., noise from nearby traffic.
- Prototype development. Starting from the premise that no user simulation can be achieved, fully developed hardware/software systems are required to validate its effectivity for navigation purposes with end-users.

In this regard, the present paper introduces Virtually Enhanced Senses (VES), an open-source 6-DoF multisensorial mixed-reality platform designed to act as a development and test-bench tool of navigation systems for BVIP. To that end, it allows for emulating various data acquisition systems, human-machine interfaces, as well as network parameters (e.g., data throughput, delay, etc.) of wireless solutions with low-cost hardware platforms.

Overall, VES is based on a highly configurable wireless sensor-actuator, a motion capture (MoCap) system compatible with the Unity game engine, and a communication protocol over UDP/IP that eases software development and allows to control low-level QoS network parameters. The prototype immerses the user in navigation scenarios with both real and virtual elements (e.g., walls, vehicles, etc.) that can be interacted with through the MoCap system, as well as haptic, acoustic, and proprioceptive feedback; all of it can be adjusted at runtime.

The system submodules will be described in the first sections, starting with the hardware platforms (Section 3.1), and followed by the wireless network protocol (Section 3.2) and motion capture system (Section 3.3) design. Finally, two experiments of random delay emulation and user navigation performance will be presented, and its results discussed, in Section 3.4, Section 4 and Section 5.

## 2. Related Work

For the last few years, there has been increasing focus on mixed-reality systems which can provide new insights in non-visual spatial perception and cognition, and particularly in the field of sensory substitution devices (SSD).

One initial example would be Chebat et al. experiments on spatial knowledge transfer obtained in real-world mazes and virtual replicas by means of the EyeCane [9], an SSD that maps one-point distance measurements to haptic output. Similarly, Lahav et al. studied navigation capabilities of blind and visually impaired people (BVIP) in a virtual environment; distance data and material composition of the virtual elements was encoded as haptic-acoustic stimuli.

However, exploring a virtual scenario while staying in a supine position sacrifices vestibular, proprioceptive, and motor feedback obtained in real-world environments. In turn, this has a negative impact on the content and accuracy of the resulting spatial knowledge and navigation performance [10]. Therefore, it motivated the development of “walkable” virtual environments with an increasing level of sensory immersion.

Recent mixed reality projects for BVIP resorted to walk-in-place solutions [11], some of which were based on treadmills [12], whereas others included products such as HTC Vive to capture 6-DoF movements of the user within a restricted area [13,14]. Conversely, Simultaneous Localization and Mapping was also used to keep track of the users’ 6-DoF head movements, emphasis placed on visual inertial odometry [15,16]. This solution supported outdoor/indoor scenarios without in-situ installation.

In addition to providing users with feedback related to their own movements, the motion capture (MoCap) systems allowed us to gather objective test data. For example, it was used to measure users’ heading and distance estimations to nearby objects or landmarks [17], or to monitor the route followed in navigation tests [15,18]. Occasionally, this was complemented by sensors that monitored physiological data such as heart rate or electrodermal activity as stress indicators along the route [19]. In parallel, spatial perception and cognition experiments with normal-sighted individuals took advantage of eye-tracking to study landmark salience [20].

In this regard, our proposed solution, VES, is a hardware/software platform based on a wireless sensor/actuator network which allows us to easily implement and validate previous and novel navigation system designs; thus far, no similar systems have been found in the literature. New sensors can be added to measure environmental or even physiological data. It also includes a configurable visual inertial motion capture that will be used to keep track of the user’s upper body; no prior in-situ installation is needed. In the test performed, it proved useful to analyze path patterns, as well as mobility and orientation performance based on a novel “gaze-tracking” approach in the field of sensory substitution.

The wireless design also introduces us to a novel challenge in this field: quality-of-service (QoS) requirements in terms of multisensory output. Current virtual reality systems have strict restrictions regarding latency [21], which made common wireless communication standards such as IEEE 802.11ac insufficient for such application [22]. Previous studies on this topic injected latency in real-time motion tracking data and observed its impact on user experience; e.g., [21,23]. However, their conclusions might not apply to non-visual mixed reality systems, and more specifically to those who implement state-of-art multisensory sensory substitution. As for the latter, no prior work could be found.

In relation to this, VES adds low-level emulation of network parameters such as data throughput or latency. We hypothesize that this topic will be key not only for current experimental mixed-reality platforms, but for future wireless market-available products.

## 3. Materials and Methods

In the following subsections, the system submodules (hardware and software) and the specific experimental setup will be described.

### 3.1. VES Wearable Device

To ease replication and distribution, VES was developed as a Unity-based application, which guarantees portability among OS such as iOS, Android, or Windows; and a FreeRTOS application for the ESP32 SoC, which would ease interfacing other embedded systems with VES and including new peripherals.

**Figure 1:**
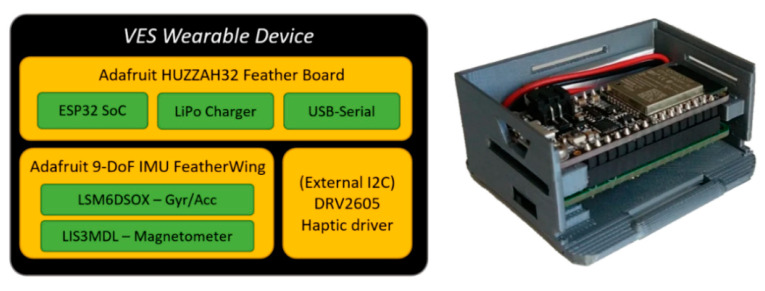
VES Wearable device and hardware architecture.

Specifically, an ESP32-based hardware platform was used as a versatile sensor/actuator node, which is shown in Figure 1. Regarding its wireless connectivity, it supports IEEE 802.11 b/g/n WiFi and Bluetooth v4.2 standards.

The current configuration includes an inertial measurement unit (IMU) for motion capture; also, a haptic driver and a simple eccentric rotating mass (ERM) motor were added, although in a separated case to avoid noise in the IMU measurements. Finally, Adafruit’s FeatherWing board includes a JST PH connector (I2C), which will be used to add future sensors/actuators.

### 3.2. Wireless Communication Protocol

#### 3.2.1. Overview

**Figure 2:**
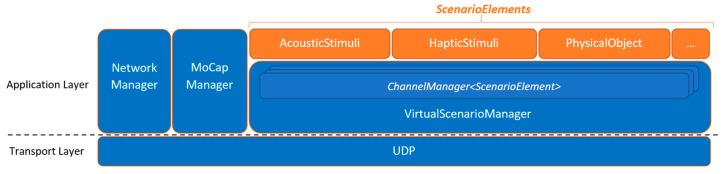
Communication protocol.

The present protocol (Figure 2) provides a software abstraction layer based on remote procedure calls that allows us to easily share synchronized sensor input and actuator output control among the wireless network; control the tasks executed by each end-device; and emulate jitter and packet loss.

The protocol was developed over UDP/IP to ease portability and to allow remote user testing in future versions; also, a connectionless protocol was needed to avoid retransmissions which would incur in added delay.

As for the role of each module:

- Network Manager: manages node discovery and monitoring (e.g., battery charge levels, availability of sensors), as well as clock synchronization. The latter simply resent clock timestamps to each device until the roundtrip time falls under a fixed threshold, by default 10 ms approx. If needed for remote tests, future versions would use standard synchronization protocols such as NTP.
- Virtual Scenario Manager: it controls the creation, destruction, and synchronization of all the objects that make up the shared virtual scenario, i.e., scenario elements (Figure 2). Further information will be included in following subsection.
- Motion Capture Manager: it handles the calibration process, including the IMU zero-input offset, as well as the forward kinematics. Thereafter, each MoCap device shares position and/or rotation data of the corresponding body part as scenario elements.

#### 3.2.2. Shared Virtual Scenario

This is a distributed data structure of the virtual environment that the user is immersed in, constructed by all the connected devices. Its design is object-oriented, where all elements derive from a “scenario element” parent class.

The implemented elements range from physical objects to haptic and acoustic feedback. Not limited to this, end-device processing tasks, e.g., by sensor input-actuator output mapping, can be modelled as scenario elements. This would ease the development of future edge-computing applications, thus avoiding network delays and reducing transmitted data throughput.

Each element, e.g., 3D-modelled objects, has three main properties:

- A unique ID that identifies it within the network.
- A proprietary device, i.e., host, that has permissions to modify its properties.
- A proprietary channel. In this regard, a channel synchronizes scenario element properties among a list of subscribed devices.

**Figure 3:**
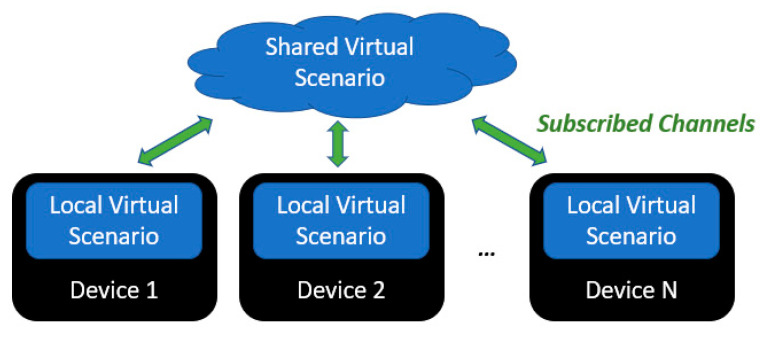
Shared virtual scenario schema.

Once a device creates an element in a specific channel, it is registered as host in a local replica of the virtual scenario (Figure 3). Thereafter, all subscribed devices creates or updates the element in their local virtual scenario as a remote element. For instance, position, orientation, or even dimensions of a physical element, e.g., a wall, can be set as properties subject to be updated throughout the network. The updates have a timestamp associated, which allows for applying at the same time in all subscribed devices. Also, it is a key feature to emulate random package delay in the communication.

#### 3.2.3. Delay and Packet Loss Emulation

Periodically, the updates of all host elements are sent to the subscribed devices. Each one of those devices identify the element’s ID and adds the update to a queue corresponding to the specific element.

These “update-queues” are sorted by its associated timestamp, i.e., the time instant in which the update must be applied. In periodic calls, each device applies all, some, or none of the available updates to each scenario element. The criterion can be set freely, and it was used to delay or discard update data according to a deterministic or random model.

The minimum period at which the data are sent and received was set as 20 ms to ease the integration with Unity engine. This was considered to be enough for most applications, starting with the developed motion capture system.

**Figure 4:**
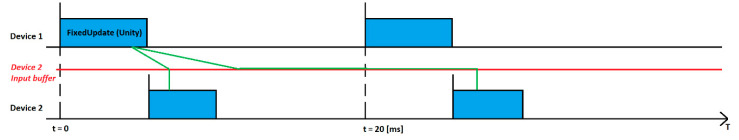
System delay example. Two devices are running a Unity-based application, which executes send/receive tasks each 20 ms. Two packages with different base delay incurs in none and 20 ms system delay.

Overall, the resulting delay is a composition of various factors grouped under the following terms. First, a base delay, defined as the time between a “send” call is made in the host device until the update data is available in a subscribed device; it aggregates delay sources which range from physical propagation through the air to the radio stack processing both at the transmitter and the receptor. Secondly, a system delay, which derives from the established update time granularity. As can be appreciated in Figure 4, this causes a random delay under 20 ms. Thirdly, and finally, our customizable delay is added.

### 3.3. Motion Capture System

As described in previous sections, VES includes a 6-DoF visual-inertial motion capture system implemented over the wireless sensor network. According to the distribution of the MoCap devices, it can be configured to capture simultaneous movements of one or more users, tools, etc.

The devices with MoCap capabilities are separated into two types:

- Visual-inertial device: an ARCore-compatible smartphone running a software application developed with Unity game engine and Google ARCore augmented reality SDK. The latter estimates the device’s position and orientation by combining camera and inertial sensor output. It also allows to fix virtual elements to specific real-world coordinates using visual markers, i.e., anchors.
- Inertial device: an embedded system with a 6-Axis IMU which provides rotation data. This role is played by VES-W devices, which include a LSM6DSL 6-Axis IMU and an ESP32 SoC. The fusing of accelerometer and gyroscope data, as well as the correspondence between IMU’s and body part’s orientation is carried out in the same device.

All position and orientation data are shared periodically, by default at a rate of 50 Hz. Once all data is gathered in a single device, it can be used to capture the motion of an articulated object. To that end, at least one visual-inertial device is required to keep track of the position and orientation in the 3-D environment; thereafter, rotation data suffices to estimate joint angles and infer the user’s pose through forward kinematics.

In the following user tests, the MoCap system was configured to record the motion of the user’s head, torso, arms, and forearms. Only one visual-inertial device is included, which was placed on the user’s head to avoid camera occlusions. Then, an inertial device was fixed to each remaining body part, making for a total of six MoCap devices.

ARCore SDK directly provides with position and orientation data; however, the orientation data from the purely inertial devices, as well as the coordination of all data prior to body pose calculations, required specific data processing from raw measurements, and a calibration method.

The orientation of each inertial device is calculated through a simple data fusion schema. Its design focuses on fast system responses based on gyroscope sensors, while the accelerometers perform long term drift corrections in the “i” and “k” axis.

Firstly, the gyroscope provides periodic rotation increments $∆r_{n}$ starting from an initial orientation quaternion $r_{n}$. Secondly, the normalized gravity vector ${\hat{g}}_{n}$ is approximated by low pass-filtered measurements of accelerometer data, ${\hat{x}}_{n}$. Thirdly, an accelerometer correction rotation quaternion $K_{acc}$ is applied, which describes the shortest arc from the IMU’s “down” vector, to the calculated gravity vector. Finally, a constant rotation $K_{offset}$ matches the orientation of the MoCap device and that of the corresponding body part.

Before normal operation, the calibration process sets an initial rotation to all MoCap devices as well as the latter rotation $K_{offset}$. To that end, three parameters need to be specified for each body part/inertial device pair: an initial orientation quaternion of the specific body part $r_{initial}$; and two reference orientation vectors that indicate the initial orientation of the inertial device in the “j” axis, one in local IMU coordinates and the other in the global coordinate system of the virtual environment.

$$r_{n}=r_{n-1}∆r_{n}\cdot {\hat{g}}_{n}=C\left(r_{n}{\hat{x}}_{n}r_{n}{}^{-1}\right)+\left(1-C\right)\;{\hat{g}}_{n-1};\;C\in \left(0,1\right)\subset \mathbb{R}$$

$$y_{n}=K_{acc}r_{n}K_{offset}\cdot K_{acc}=e^{\frac{\mathrm{acos}\left(-j\cdot {\hat{g}}_{n}\right)}{2}\left(-j\times {\hat{g}}_{n}\right)}\cdot K_{offset}=r_{n}{}^{-1}r_{initial}$$

Once the avatar is calibrated, the usage of visual anchors allows for fixing accumulated positioning errors, as well as to synchronize real and virtual elements. Also, this feature can be further enhanced through tools such as Vuforia Area Targets, which uses complete 3D scans of the surroundings as anchors.

### 3.4. Experimental Setup

**Figure 5:**
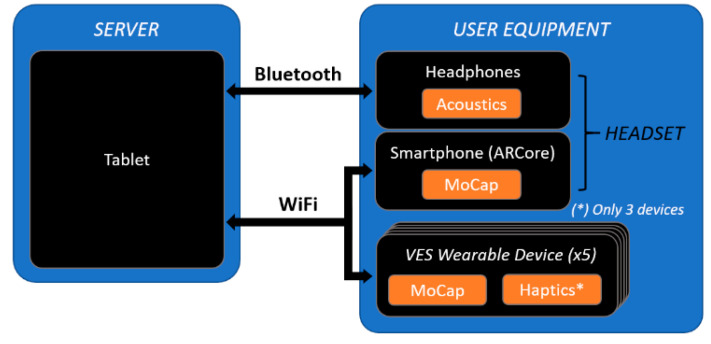
System architecture used in the tests.

The specific devices used and the role of each one in the system was set as follows (Figure 5):

- Server: A Samsung Galaxy Tab 6 tablet running a GUI interface developed with Unity game engine which renders all 3D elements of the shared virtual scenario and allows for controlling the system configuration through a GUI. Specifically, it will control the sensor input-stimuli output mapping, and the navigation environment, e.g., a previously 3D scanned room [16].
- User equipment: A Xiaomi Mi 9T Pro smartphone, five “VES Wearable Devices” (VES-W) and Bluetooth headphones. The smartphone runs a Unity-based software application which captures head movements through visual inertial odometry (ARCore) and adds it to the shared virtual scenario as a 3D object. Analogously, the VES Wearable (VES-W) devices capture and share torso, arms, and forearms motion data.

As described in previous sections, VES includes a 6-DoF visual-inertial motion capture system implemented over the wireless sensor network. According to the distribution of the MoCap devices, it can be configured to capture simultaneous movements of one or more users, tools, etc.

**Figure 6:**
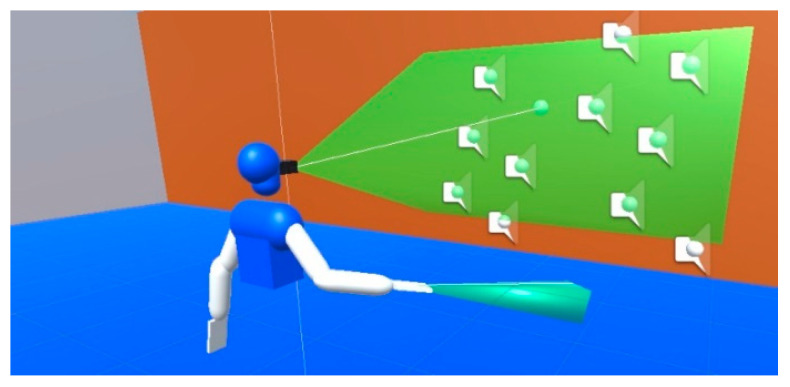
Detection areas for the acoustic (PVAS) and virtual-cane haptic feedback.

Thereafter, three sensory substitution algorithms were included as means to interact with the virtual scenario:

- Projected Virtual Acoustic Space (PVAS) [16]. It is visual-acoustic sensory substitution algorithm based on Virtual Acoustic Space [24]. The algorithm simulates a depth sensor carried by the user and generates virtual sound sources over the surfaces in range of the sensor’s field of view, as shown in Figure 6. The sensor’s range was set to 20 m, with a field of view of 50 × 30 degrees. As for the output, it reproduces a sequence of short audio pulses (typically 20–50 ms) which differs according to the object’s material.
- A minimalistic visual-haptic sensory substitution system which increases the stimuli intensity, i.e., vibration of an ERM/LRA actuator, as the object gets closer. Just like the previous algorithm, it uses a configurable detection range which can be fixed anywhere on the user’s body. In the following sections it will be used to simulate a virtual cane (Figure 6) of 1.2 m long, and short-ranged obstacle avoidance devices in both arms with 0.4 m detection radius, similar to Virtual Haptic Radar [25].
- The vOICe [8]. This is one of the first developed and most studied visual-acoustic sensory substitution, which encodes M × N low resolution grayscale images into a set of tones. Specifically, it divides each image into a set of M pixels corresponding to each column of pixels. Thereafter, each vector of N pixel values is mapped to a summatory of N tones of increasing frequency; a pixel of small index and value trigger low-intensity, low-frequency tones.

As for the current implementation, only Unity embedded tools were used. The images of a virtual camera were rendered into a configurable low-resolution image, while the output was generated through an “AudioSource” component. Based on prior results [8], The default configuration was set to a 64 × 64 image resolution; 8 grayscale pixel values; and tones exponentially distributed in frequency, from 500 Hz to 5000 Hz.

#### 3.4.1. Random Delay Emulation

Given that the sensory substitution algorithms are linked to detection areas fixed to the user’s body (previous section), a random delay of the body’s movement involves a random delay in the user’s feedback. Therefore, it results in a mismatch of the haptic, acoustic, and proprioceptive feedback which, in turn, may result in a degraded distance perception, less accurate estimation of landmark relative position, etc.

As a first approximation, only delay measurements will be analyzed in this test. The motion capture data that serves as the input of the acoustic and haptic feedback algorithms will be delayed by two independent random variables, one for each sensor modality. Specifically, the probability of applying “*k*” number of updates in a single call follows the expression:

$$f\left(k,\lambda ,n\right)=\min\left(n,\;\frac{e^{-\lambda }\lambda^{k}}{k!}\right);\;n,\;k\in \mathbb{N}\;;\;\lambda \in {\mathbb{R}}^{+}$$

where “λ” is the average number of packages updated per call, and “*n*” is the length of the scenario element buffer. As can be seen, this corresponds to a truncated Poisson distribution, with a maximum output per call set to “*n*”.

The system configuration detailed in the previous section was applied, and “λ” was set to 1.2 and 1.5 for the acoustic and haptic feedback, respectively. The user will then move for five minutes (5000 update packages), and the system will record the MoCap data timestamps of the right arm, its base delay, and the added delay of the data provided to the acoustic/haptic sensory substitution algorithms.

#### 3.4.2. User Orientation and Mobility Performance

This experiment serves a double purpose. Firstly, it allows for checking if VES simulation of a navigation system in a virtual scenario can be understood and used by a test subject, although it is not enough by itself to guarantee equivalence of user performance between the simulated and the actual system. In this regard, we hypothesize that the motion capture system plays a key role (Section 5). Secondly, it shows how the output of the system can be used to analyze user behavior.

Given the objectives of this first stage of prototype validation, it was considered that normal-sighted blindfolded individuals sufficed. In total, 23 subjects were tested, out of which 18 were female, aged between 20 and 59 (average 40).

**Figure 7:**
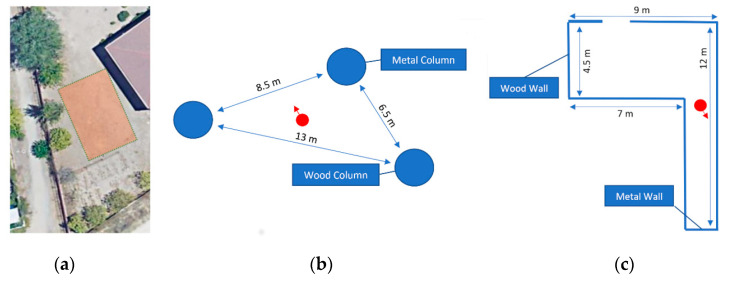
Testing field (**a**). Virtual environment of the first (**b**) and second (**c**) tests. The starting position and orientation are represented by a red dot and arrow.

The experiment was divided in two separate tests, in which the user will be asked to reach specific locations in two virtual environments loaded in an approximately 12 × 30 outdoor field (Figure 7). The objective was to assess whether the users can interact with the virtual environment generated by VES and make use of the provided spatial feedback to perform basic orientation and mobility tasks.

Prior to the tests, the users conducted a training session to get familiarized with the haptic/acoustic feedback. The users were equipped both with the prototype and the server/tablet, so they can see both its own movements and the range of the virtual sensors that trigger haptic/acoustic stimuli. Thereafter, they were immersed in a hallway of 5 × 1 m, and were asked to walk from one end to the other and return. Finally, they had to repeat the route blindfolded.

The environment of the first test was composed of three columns with a diameter of 2 m; each one is made of a different “acoustic” material. The users were told that there are surrounded by three columns, but they did not know of their starting position and orientation, nor the relative position of those columns. Then, they are asked to “touch” them with the virtual cane in succession, clockwise. The test was considered passed if the user touched all three columns within 5 min.

The second test took place in a virtual room, the same that was used in a previous publication [16]. The virtual cane was disconnected; therefore, the only haptic feedback that remained was that of the short-ranged (0.4 m) obstacle detection sensors of the arms. All users were shown a map of the room, including wall composition. Then, they were positioned in an unknown starting point, and were asked to get out through the aperture, i.e., door. The time limit was fixed to 3 min.

All movements of the users were recorded in JSON files, which contains the position vector and orientation quaternion of each body part each 20 ms. Although this format is not efficient in terms of size, it can be imported in a variety of data processing tools. Analogously, the “collision” points of the virtual sensors which triggered virtual and acoustic stimuli were included in separate JSON files.

Thereafter, MATLAB was used to analyze patterns in the travelled path, and a representation of which elements entered the detection range of the haptic-acoustic feedback more frequently. The latter will be a first approach to “gaze-tracking” in the field of sensory substitution, which is hypothesized to be useful in the study of landmark salience in orientation tasks.

## 4. Results

### 4.1. Random Delay Emulation

**Figure 8:**
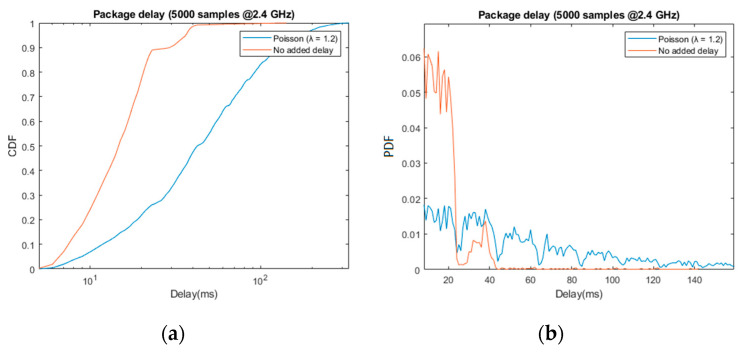
Capture of the update data’s delay before (orange) and after (blue) adding jitter, λ = 1.2. The graphs show the cummulative distribution function (**a**) and the probability distribution function (**b**) of the sample (5000 packages sent).

**Figure 9:**
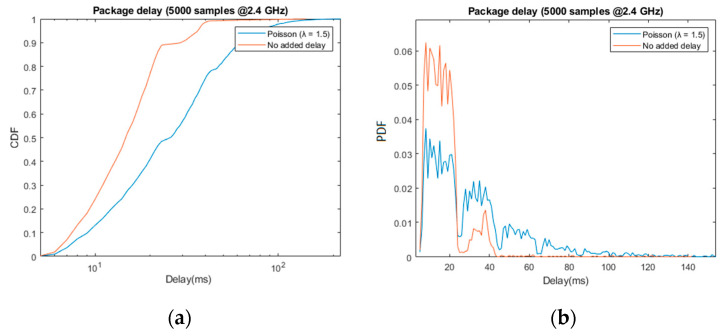
Capture of the update data’s delay before (orange) and after (blue) adding jitter, λ = 1.5. The graphs show the cummulative distribution function (**a**) and probability distribution function (**b**) of the sample (5000 packages sent).

In the following graphs, we include delay measurements corresponding to the transmitted update packages, before and after adding random transmission delay. The lost packages, which amount to a total of 1.4% of those transmitted, were discarded. The Table 1 and the first two graphs (Figure 8) refer to the input of the acoustic feedback algorithm analogously, while the latter two graphs (Figure 9) refer to the input of the haptic feedback algorithm.

### 4.2. User Orientation and Mobility Performance

The MoCap measurements recorded in the experiment provide a wide range of data: the number of collisions per user; heading angles towards specific landmarks, e.g., the columns of the first test; instantaneous walking speed; delay between an obstacle-detected haptic warning is triggered and a hypothetical response in terms of walking speed; etc. The raw data is available in [26]. However, as stated in the “methods” section, only the following results will be discussed: 2-D representations of the most-travelled paths, and the most-perceived elements of the scenario.

**Figure 10:**
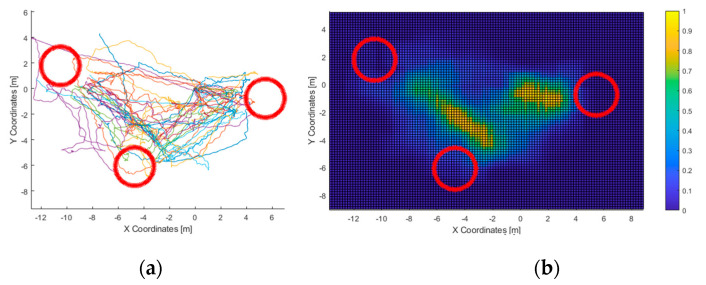
Test 1—Route followed and path heatmap. All routes overlapped (**a**) and the corresponding most-frequented areas heatmap (**b**). As for the latter, the data was obtained with a spatial resolution of 5 cm, and a low-pass FIR of 25 × 25 cell flat impulse response.

As for the first, all data paths were combined in a 2-D histogram, low-pass filtered, and finally, normalized. This results in a “heatmap” of the most-frequented areas, i.e., path heatmap (Figure 10). Thereafter, the surfaces which triggered haptic/acoustic stimuli are considered: the accumulated time in which they generated feedback is represented in separated, normalized 2D histograms. From now on, these will be referred as haptic and acoustic heatmaps.

**Figure 11:**
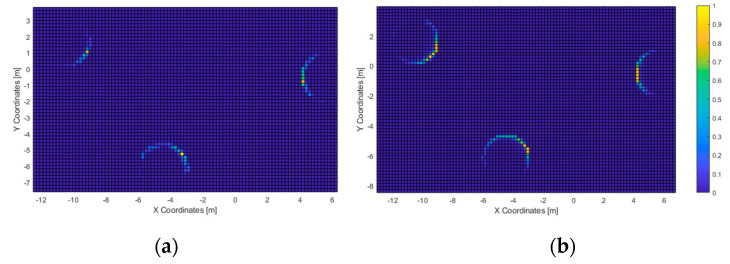
Test 1—Haptic (**a**) and acoustic (**b**) heatmaps. Spatial resolution: 20 cm.

In the first test, all users touched all three columns within the time limit. The average time needed was of 135 s, with a minimum value of 59 and a standard deviation of 63. The routes followed and the corresponding heatmap is represented in Figure 10, followed by the heatmaps of the most-perceived elements (Figure 11).

**Figure 12:**
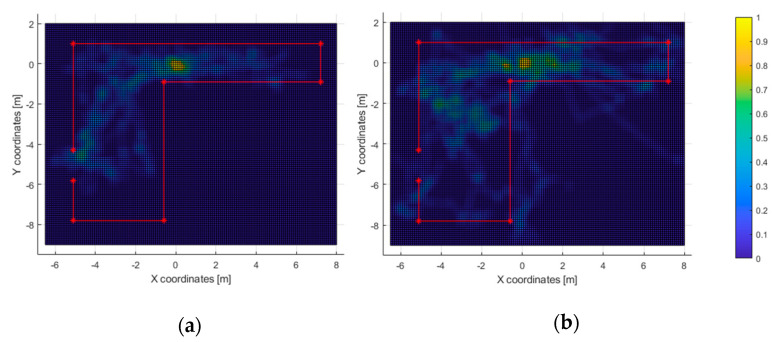
Test 2—Path heatmaps of those who passed the test (**a**) and those who did not (**b**). Spatial resolution: 5 cm. Low-pass FIR: 9 × 9 cell flat impulse response.

**Figure 13:**
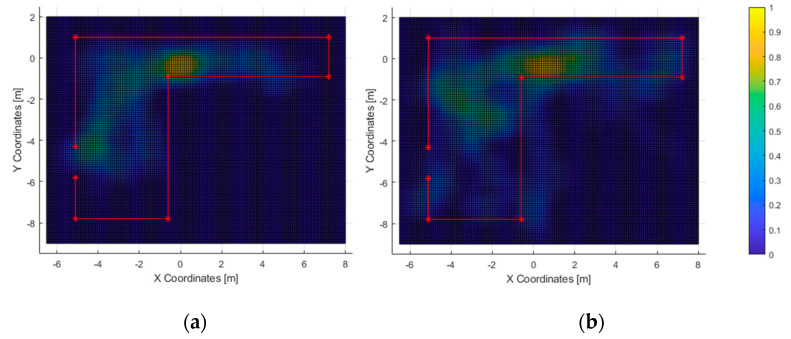
Test 2—Path heatmaps of those who passed the test (**a**) and those who did not (**b**). Spatial resolution: 5 cm. Low-pass FIR: 25 × 25 cell flat impulse response.

**Figure 14:**
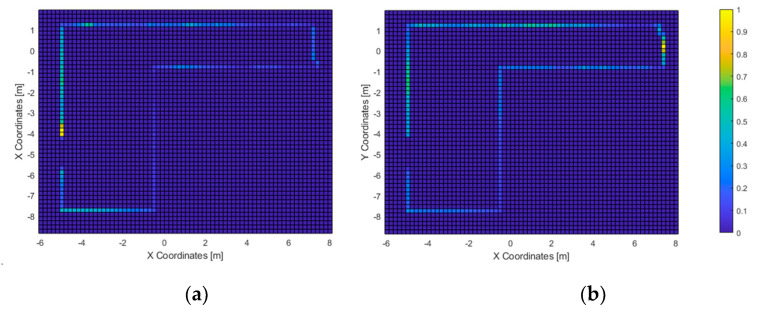
Test 2—Acoustic heatmaps of those who passed the test (**a**) and those who did not (**b**).

**Figure 15:**
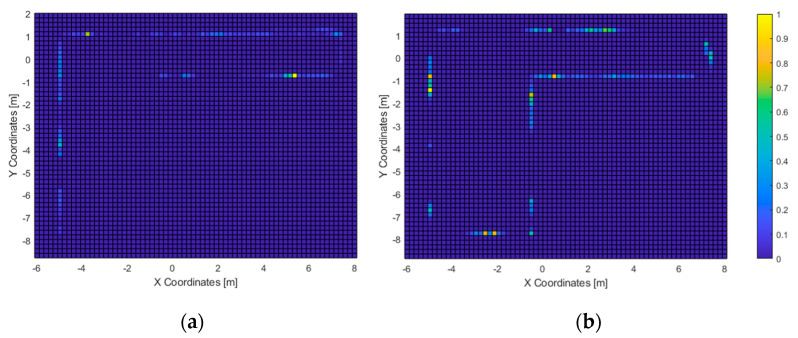
Test 2—Haptic heatmaps of those who passed the test (**a**) and those who did not (**b**).

In the second test, 11 out of 23 subjects were able to exit the room (approx. 48%) within the time limit, with an average completion time of 136 s and a minimum of 75 s. The heatmaps of the most-travelled areas are displayed in Figure 12 and Figure 13. Next, the heatmaps of the most-perceived elements are included in Figure 14 and Figure 15.

## 5. Discussion

Starting with the random delay emulation, the results obtained are coherent with the design described in Section 3.2.3: approximately 90% of the received packages arrived within the first 25 ms period, and 96% before 37 ms. The second peak shown in the normalized histogram, i.e., pdf, as well as the harmonic-like pattern in the total delay (blue), confirming the effect of the periodicity in the data reception.

Even in the best-case scenario, these results are worse than standard VR solutions over WiFi, which can offer average delays under 2 ms (e.g., 802.11ac in [22]). However, it suffices to provide mobility and orientation assistance, as can be appreciated in the resulting mobility and orientation performance of both tests.

In the first test, all users were able to “touch” all three columns consecutively. Given that the haptic feedback is short ranged (the virtual cane had a length of 1.2 m), the acoustic feedback was used by all users to recognize all the elements in the scenario. This matches with the acoustic heatmap (Figure 11): the columns did trigger feedback. Furthermore, they were “seen” from the area at the center of the virtual scenario, in accordance with the path-travelled heatmap.

Not limited to this, the path-travelled heatmap (Figure 10) shows the starting point as well as the most-frequented route: it starts with the column at the right side of the graph, followed by the one at the bottom, and finally, the one in the left. It is composed mainly of straight lines that stopped at a fixed distance from the columns. This accounts for the accuracy of heading estimations from the 3D virtual sound sources generated by VES; and, considering the haptic heatmap, for the usefulness of the simulated virtual cane to avoid collisions with the virtual elements of the environment.

In the second test, nearly half of the users were able to exit the room within the time limit. Those who passed the test tended to walk directly to the exit (Figure 13a). Also, the path heatmap is adjusted to the shape of room; it can be assumed that the users detected the virtual walls as such and avoided them.

The acoustic heatmap (Figure 14a) suggest that the wood wall at the bottom of the figure was used as a key landmark, as well as the one on the left where the door is located. Conversely, the haptic heatmap (Figure 15a) shows that the obstacle-avoidance haptic warnings were triggered all along the hallway and near the left wall. This matches with the high-resolution heatmap of Figure 12, as the paths in the hallway are close to the walls. Also, it can be noticed that some users made use of this short-ranged feedback to follow the route all along the wall to the exit.

As for the ones who did not complete the second test, the high-resolution path heatmap of Figure 12b and the corresponding haptic heatmap (Figure 15b) exhibit a tendency of walking through all the virtual walls. Even then, the low-resolution path heatmap (Figure 13) shows a tendency to stay within the room. Also, the acoustic heatmap shows that some test subjects observed the end of the hallway as well as the left and top walls. Taking in consideration the high dispersion in the path heatmap near the starting point, it suggests a large search for landmarks right after the test began.

These results, however, are limited by the capability of VES to simulate a fully developed navigation system. In this regard, we started from the premise that two systems are equal if, when facing the same input conditions, both provide the same output. The input of the simulated system is generated from previously registered data of the environment and the motion capture system. Therefore, we conclude that the specifications of the latter are the decisive factor that guarantees equivalence of the simulated and the actual system and, therefore, of the resulting user performance.

Accordingly, the next step prior to future end-user testing will be a characterization of the current MoCap system. At this point, we emphasize that the accuracy, latency or reliability requirements of the motion capture system depends on the specific navigation system to be implemented, as discussed in a previous publication [2]. For instance, only meter-level accuracy positioning is needed for GNSS route guidance solutions, while the usage of 3D sounds involves binaural output which usually require cm accuracy positions and split-second time responses. Nevertheless, if the requirements to simulate a specific navigation system exceeds those of the developed solution, it can be enhanced, e.g., by including Vuforia Area Targets; or replaced, taking advantage of VES modular design.

Next, some brief points will be discussed in relation with VES supported navigation systems and the corresponding hardware requirements.

Firstly, “The vOICe” exemplifies how images of a virtual camera can be processed in VES to generate an auditory output in execution time, which is applicable to SSD such as SmartSight [27]. Furthermore, given that the ARCore-compatible smartphone captures both position and rotation of the users’ head, it suffices to calculate the pose of the virtual camera and to generate the acoustic output. Analogously, if an appropriate haptic interface is included, e.g., an array of haptic actuators or an electrotactile display, recent visual-tactile SSD systems based on Bach-y-Rita pioneer TVSS [28] like BrainPort [7], Forehead Retina System [29] or HamsaTouch [30] can also be integrated.

Secondly, the usage of virtual proximity sensors and upper-body MoCap allowed to implement PVAS and a virtual cane, which relates to SSD like ENVS [31] or EyeCane [32], as well as market-available obstacle avoidance systems such as Sunu Band [33].

Thirdly, in addition to SSD, several systems used virtual sound sources to intuitively guide users along a route, e.g., [34,35]. As appreciated in PVAS implementation and the corresponding user tests, these acoustic cues are supported by VES; in fact, these were the only cues available to reach the three columns in the first VES navigation experiment (Figure 10 and Figure 11b).

If integrated in VES, these systems would benefit from the usage of previously modelled information, thus allowing for isolating the design of the human-machine interface from the data acquisition system. This could play a key role to reduce the information provided by previous SSD, e.g., background noise; reduce cognitive load; and ease the identification of surrounding elements, e.g., by highlighting the difference between figure and background.

Finally, although the tests were conducted in purely virtual scenarios, VES allows to integrate virtual and real scenario elements, sensors, etc. By means of visual anchors, the movements of the user in a real environment can be matched with those of the avatar in a virtual replica. Thereafter, real and simulated systems, as well as a cane or other medium to interact with the environment, can be used simultaneously.

## 6. Conclusions

The VES system was validated as a novel, low-cost development and test-bench tool of navigation systems for the visually impaired. From the experiments conducted, we inferred that sensory substitution algorithms such as “The vOICe” or the presented PVAS could be tested with only an ARCore-compatible smartphone, headphones, and a server which runs the GUI. Also, the popular ESP32 SoC served to add a configurable number and type of peripherals to the mixed-reality platform. Specifically, haptic actuators and IMU sensors were integrated and used to implement a simple “Virtual cane” attached to the user’s right forearm, as well as obstacle-detection ETAs fixed to both arms.

Additionally, the present work introduces two new tools which could be key in future development of non-visual human-machine interfaces: an adaption of gaze-tracking techniques to sensory substitution experiments; and low-level emulation of network QoS, useful to test the user requirements in terms of multisensory feedback synchronization.

## Figures and Tables

**Table 1:** Delay statistics of the update packages.

	Minimum	Maximum	Average	Std. Deviation
Base delay	5	142	17	10
Total delay (λ = 1.5)	5	330	60	52
Total delay (λ = 1.2)	5	220	32	24

## Data Availability

The raw results of the user tests are available online at ‘http://elb105.com/ves/’, accessed on 13 September 2021.
